# The sensitivity of acute myeloid leukemia cells to cytarabine is increased by suppressing the expression of Heme oxygenase-1 and hypoxia-inducible factor 1-alpha

**DOI:** 10.1186/s12935-024-03393-3

**Published:** 2024-06-25

**Authors:** Mohammad Sadeghi, Asma Moslehi, Hadiseh Kheiry, Fariba Karoon Kiani, Asieh Zarei, Atefeh Khodakarami, Vahid Karpisheh, Ali Masjedi, Badrossadat Rahnama, Mohammad Hojjat-Farsangi, Mortaza Raeisi, Mehdi Yousefi, Ali Akbar Movasaghpour Akbari, Farhad Jadidi-Niaragh

**Affiliations:** 1 Immunology Research Center, Tabriz University of Medical Sciences, Tabriz, Iran; 2 Student Research Committee, Tabriz University of Medical Sciences, Tabriz, Iran; 3 Institute of Experimental Hematology, School of Medicine, Technical University of Munich, 81675 Munich, Germany; 4 Center for Translational Cancer Research (TranslaTUM), School of Medicine, Technical University of Munich, 81675 Munich, Germany; 5 Department of Oncology-Pathology, Karolinska Institute, Bioclinicum, Stockholm, Sweden; 6 Department of Immunology, Faculty of Medicine, Tabriz University of Medical Sciences, Tabriz, Iran; 7 Hematology and Oncology Research Center, Tabriz University of Medical Sciences, Tabriz, Iran

**Keywords:** Cancer immunotherapy, AML, HO-1, HIF-1α, Cytarabine, Leukemia

## Abstract

**Background:**

Acute myeloid leukemia (AML), a malignancy Often resistant to common chemotherapy regimens (Cytarabine (Ara-c) + Daunorubicin (DNR)), is accompanied by frequent relapses. Many factors are involved in causing chemoresistance. Heme Oxygenase-1 (HO-1) and Hypoxia-Inducible Factor 1-alpha (HIF-1α) are two of the most well-known genes, reported to be overexpressed in AML and promote resistance against chemotherapy according to several studies. The main chemotherapy agent used for AML treatment is Ara-c. We hypothesized that simultaneous targeting of HO-1 and HIF-1α could sensitize AML cells to Ara-c.

**Method:**

In this study, we used our recently developed, Trans-Activator of Transcription (TAT) - Chitosan-Carboxymethyl Dextran (CCMD) - Poly Ethylene Glycol (PEG) - Nanoparticles (NPs), to deliver Ara-c along with siRNA molecules against the HO-1 and HIF-1α genes to AML primary cells (ex vivo) and cell lines including THP-1, KG-1, and HL-60 (*in vitro)*. Subsequently, the effect of the single or combinational treatment on the growth, proliferation, apoptosis, and Reactive Oxygen Species (ROS) formation was evaluated.

**Results:**

The designed NPs had a high potential in transfecting cells with siRNAs and drug. The results demonstrated that treatment of cells with Ara-c elevated the generation of ROS in the cells while decreasing the proliferation potential. Following the silencing of HO-1, the rate of apoptosis and ROS generation in response to Ara-c increased significantly. While proliferation and growth inhibition were considerably evident in HIF-1α-siRNA-transfected-AML cells compared to cells treated with free Ara-c. We found that the co-inhibition of genes could further sensitize AML cells to Ara-c treatment.

**Conclusions:**

As far as we are aware, this study is the first to simultaneously inhibit the HO-1 and HIF-1α genes in AML using NPs. It can be concluded that HO-1 causes chemoresistance by protecting cells from ROS damage. Whereas, HIF-1α mostly exerts prolific and direct anti-apoptotic effects. These findings imply that simultaneous inhibition of HO-1 and HIF-1α can overcome Ara-c resistance and help improve the prognosis of AML patients.

**Supplementary Information:**

The online version contains supplementary material available at 10.1186/s12935-024-03393-3.

## Introduction

Acute myeloid leukemia (AML) is a hematological neoplasm, resulting from the clonal proliferation of malignant myeloid progenitors [1, 2]. AML is distinguished by the repletion of myeloblasts in the Bone Marrow (BM) and the Peripheral Blood (PB) [3]. The treatment may include chemotherapy and Hematopoietic Stem Cell Transplantation (HSCT) [2]. The main chemotherapy regimens for AML consist of Cytarabine (Ara-c) plus Daunorubicin (DNR) [4]. Despite these intensive treatment regimens, overall and disease-free survival rates of patients remain low due to frequent relapse and chemo-resistance, in particular for the elderly [2, 5, 6]. Thus, identifying new therapeutic targets for AML is of great importance [2].

Ara-c, a pyrimidine nucleoside analog, is one of the major AML treatment regimens, which is injected intravenously. Following cellular uptake, Ara-c is metabolized into nucleotide triphosphate by deoxycytidine kinase and is incorporated into the DNA strand, resulting in cell cycle arrest in the S phase [7, 8]. Moreover, Ara-c is reported to induce cell death by inducing the formation of Reactive Oxygen Species (ROS). ROS generation subsequently upregulates antioxidant enzymes such as Heme oxygenase 1 (HO-1). Therefore, silencing HO-1 can be beneficial by further sensitizing AML cells to Ara-c [9].

HO-1 or Heat shock protein 32 (Hsp32) is an inducible enzyme coded by the HMOX1 gene on the 22q13.1 chromosome [10]. HO-1 catabolizes heme (a prosthetic group of hemoproteins, and an oxidant) into biliverdin, iron, and carbon monoxide [11]. According to several studies, HO-1 is overexpressed in different malignancies [2]. HO-1 protects leukemic cells by various mechanisms such as inducing autophagy [12], proliferation, inhibiting inflammation [13], and apoptosis in response to chemotherapeutics [1]. HO-1 utilizes various pathways and interacts with several molecules to protect leukemic cells, but not all of them are known.

**Fig. 1:**
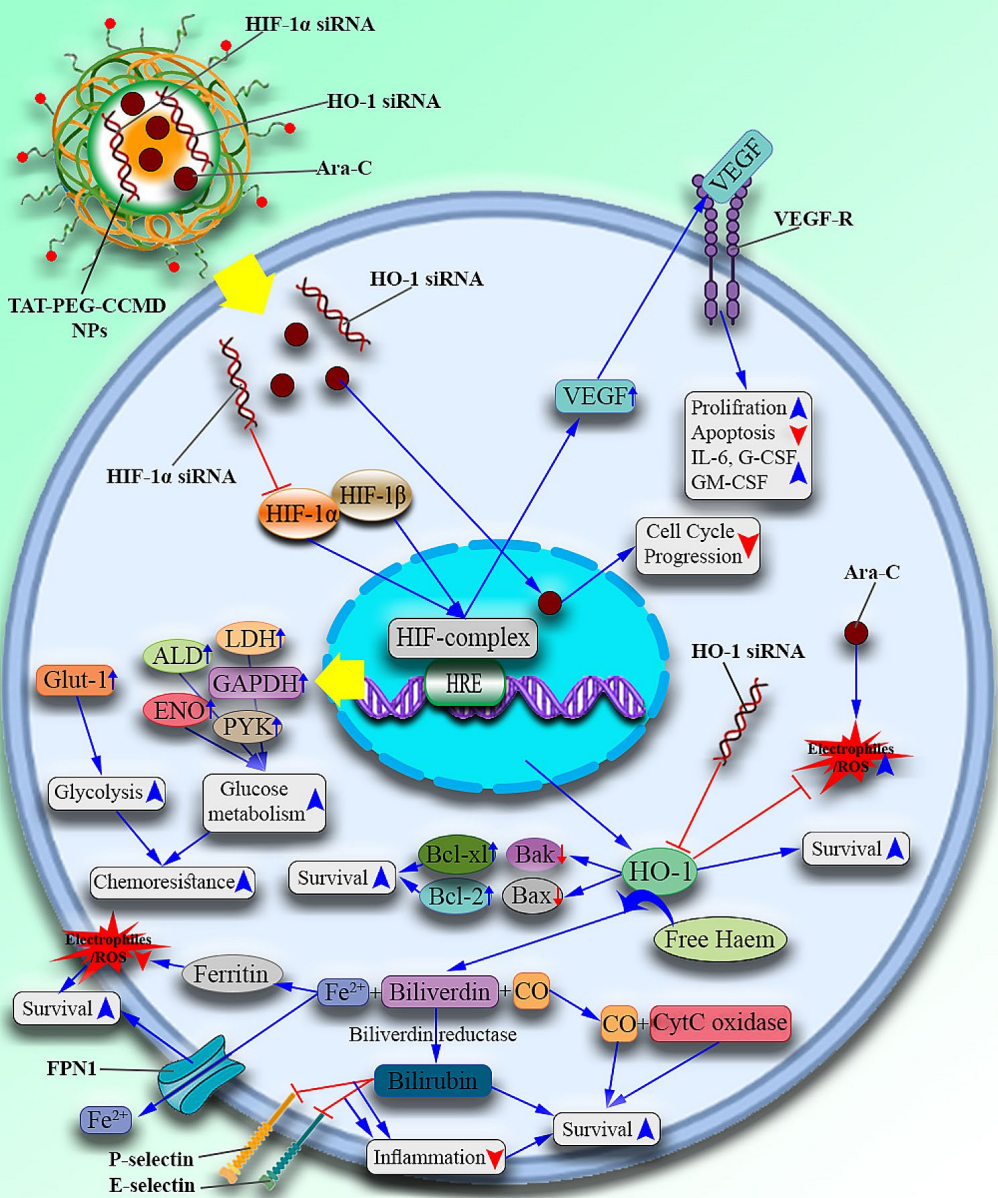
The co-inhibition of HO-1 and HIF-1α synergizes with Ara-c treatment in AML. HIF complex is made of two subunits: HIF-1α and HIF-1β. Under atmospheric conditions, HIF-1α is hydroxylated on proline residues by Prolyl hydroxylases (PHDs), followed by binding to Von Hippel-Lindau (VHL), resulting in HIF-1α ubiquitination and degradation by the proteasome. While in hypoxic conditions, HIF-1α is preserved from degradation. In AML cells, HIF-1α is aberrantly overexpressed, despite the absence of hypoxia. Several molecular mechanisms have been proposed to explain this phenomenon (described in the discussion). Following the accumulation of HIF-1α and HIF-1β in the nucleus, the HIF-1 complex is formed which then binds to the HRE regions on the promotors of HIF-1 target genes. These genes include HO-1, VEGF, ENO, GAPDH, ALD, PYK, GLUT-1, LDH, etc. involved in the chemoresistance of AML cells. The induced expression of GLUT-1 by HIF-1 is reported to induce glycolysis and hypoxia resistance resulting in AML chemoresistance. The upregulated ALD, ENO, LDH, PYK, and GAPDH elevate glucose metabolism and induce chemoresistance as well. HIF-1 complex is also reported to induce the production of VEGF, which in turn induces proliferation and inhibits leukemic cell apoptosis by binding to its receptor. Also, this binding induces IL-6, GM-CSF, and G-CSF production resulting in further proliferation. Another target gene of the HIF-1 complex is HO-1. HO-1 exerts direct anti-apoptotic effects on the cell by upregulating Bcl-XL, and Bcl-2, and downregulating BAX and BAK. It is also reported that this molecule increases cell survival by inducing the JNK-c-Jun signaling pathway. However, almost all of the anti-apoptotic actions of HO-1 are derived from its products. HO-1 is responsible for the degradation of free heme into Fe^2+^, CO, and biliverdin. By degrading free heme molecules of the cell, HO-1 decreases ROS production and cell damage, as free heme is considered a crucial factor leading to the production and accumulation of ROS in the cell. Released CO molecule binds to Cyt C Oxidase which in turn activates the p38 MAPK signaling pathway, resulting in cell survival. Biliverdin is converted into bilirubin (a strong anti-oxidant) and protects the cell from damage caused by ROS. Also, bilirubin possesses anti-inflammatory properties as well, due to its ability to inhibit the presence of P and E selectins on the surface of the cell. The released Fe^2+^ atom induces ferritin production as well as the activity of a Fe^2+^ efflux pump called FPN1, both of which result in decreased ROS production and improved cell viability. Therefore, by simultaneous inhibition of these genes combined with a more efficient Ara-c delivery into the cells, the pro-apoptotic activity of Ara-c is improved and the amount of ROS in the cell is predicted to be elevated, resulting in more apoptosis

Hypoxia-Inducible Factors (HIF) are super-regulators in several processes such as angiogenesis and proliferation [14]. Our recent experiences have shown that targeting HIF-1 has a significant effect in inhibiting the growth and spread of cancer [15, 16]. HIF-1α is a ubiquitously expressed subunit and is reported to be overexpressed in AML cells [17]. HIF-1α induces Glucose Transporter-1 (GLUT-1), and hexokinase expression. Therefore, resulting in glycolysis induction, rather than in oxidative phosphorylation, thus decreasing ROS generation [1, 18]. HIF-1α also upregulates Vascular Endothelial Growth Factor (VEGF) as well as CXC chemokine type 4 Receptors (CXCR4) [18]. It has been reported that following HO-1 inhibition by siRNA, HIF-1α expression was also decreased [1]. Thus, it seems that HO-1 influences HIF-1α expression as well. Resistant leukemic cells are also reported to be adapted to hypoxic conditions as well [7, 19]. Thus, HIF-1α, as a major response element to hypoxia, might be a suitable target. HO-1 and HIF-1α are reported to be overexpressed in AML cells [2, 20]. Various roles of HIF-1α and HO-1 in AML resistance to therapy are demonstrated in Fig. 1.

In the current study, siRNA molecules were used to silence genes and study their functions. Although siRNAs can inhibit gene expression, they are poorly unstable due to degradation by phosphatases and RNases [21]. To efficiently transfect cells with siRNA and increase the stability of siRNAs, we used our recently developed nanoparticles (NPs) [22]. NPs increment the solvency of medications and diminish the necessary successful dose [23, 24]. We used Trans-Activator of Transcription (TAT)-Chitosan-Carboxymethyl Dextran (CCMD)-Poly Ethylene Glycol (PEG) NPs designed in our previous study. TAT usage increases the efficiency of transfection. All the necessary tests on NPs to ensure the efficient delivery of the therapeutics to the leukemic cells were performed in our previous study [22].

As far as we are aware, there is no similar research targeting both HO-1 and HIF-1α simultaneously in AML using NPs. In the following study, the effect of Ara-c on ROS generation as well as the proliferation of AML cell lines was studied. In addition, HO-1 and HIF-1 inhibition effect combined with Ara-c was investigated on the growth of AML cells.

## Methods

### Reagents

Ara-c was obtained from Cayman Chemical Company. NaOH was purchased from Sigma-Aldrich (Manheim, Germany). Human HIF-1α targeting siRNA (catalog number: sc-35,561), and human HO-1 targeting siRNA (catalog number: sc-35,554), were bought from Santa Cruz Biotechnology, Inc. The 3-(4,5-dimethylthiazol-2-yl)-2,5-diphenyl-2 H-tetrazolium bromide (MTT) cell proliferation assay kit was purchased from Sigma Aldrich and was used according to the directions given by the company. Cell Proliferation Enzyme-Linked ImmunoAssay (ELISA) bromodeoxyuridine (BrdU) (colorimetric) was supplied by (Roche Molecular Biochemicals, Indianapolis, IN).

### Clinical samples and cell lines

After obtaining informed consent from patients, heparinized PB and BM samples were collected from 11 Untreated/ relapsed AML patients from Shahid Ghazi hospital in Tabriz according to the declaration of Helsinki. A brief description of patients’ characteristics is presented in the supplementary Table S1. Peripheral blood mononuclear cells (PBMCs) and bone marrow mononuclear cells (BMMCs) were isolated from the samples via Ficol paque Plus (Sigma Aldrich). HL-60 (AML-M2), THP-1 (AML-M5), and KG-1 (AML-M6) cell lines were supplied by the national cell bank of Iran (Pasteur Institute of Iran). Cells were cultivated in RPMI-1640 medium, enriched with 20% FBS, 100 unit/mL streptomycin, and 100 unit/mL penicillin, and cultured in a standard incubator with 95% humidity, 37 °C temperature, and 5% CO_2_.

### Transfection of cells with NPs loaded with siRNA and Ara-c

In the current study, CCMD-PEG-TAT NPs were used to deliver Ara-c and siRNAs against HO-1 and HIF-1 into the leukemic cells. As demonstrated by our previous study, these NPs are highly effective in transfecting hematological malignant cells [22]. The presence of TAT increases the efficiency of this transfection [22]. The cells were cultivated and then treated with one of the following pharmacological compounds for 24 (for qRT-PCR, MTT test), 48 h (for MTT test), and 72 h (for protein analysis); Untreated, NP-HIF-1α siRNA, NP-HO-1 siRNA, NP-HO-1 + HIF-1α siRNA, Ara-c, NP-Ara-c, NP-HO-1 siRNA-Ara-c, NP- HIF-1α siRNA-Ara-c, NP- HIF-1α + HO-1 siRNA + Ara-c. For each treatment group, 50 nM of each siRNA was used. The amount of drug was used according to the measured IC_50_ value for each cell (Supplementary Figure S1 and Table 1).

**Table 1:** The IC_50_ values of cell lines and primary cells

Cell type	IC_50_(µM)
HL-60	14.24
KG-1	18.21
THP-1	23.2
PBMC	11.48
BMMC	10.29

### Gene expression analysis

The total RNA of cells was extracted by TRIzol Reagent (Invitrogen). RNA was then transcribed into cDNA and stored at -20 °C. Using SYBER Green PCR Master Mix (Thermo Fisher, US) a µl of cDNA was used for qRT-PCR by a light-cycler 480 qRT-PCR system (Roche). ∆∆CT method was utilized for quantification relative to β-actin gene expression. The used primer sequences are available in Table 2.

**Table 2:** Primer sequences

gene	Primer type	sequence	Reference
*HO-1*	Forward	5’-ACCCATGACACCAAGGACCAGA-3’	[25]
	Reverse	5’-GTGTAAGGACCCATCGGAGAAGC-3’	
*HIF-1α*	Forward	5′- TATGAGCCAGAAGAACTTTTAGGC − 3′	[26]
	Reverse	5′- CACCTCTTTTGGCAAGCATCCTG − 3′	
*Bcl-2*	Forward	5’- TCGCCCTGTGGATGACTGA − 3’	[27]
	Reverse	5’- CAGAGACAGCCAGGAGAAATCA − 3’	
*BAX*	Forward	5’- CCCGAGAGGTCTTTTTCC − 3’	[28]
	Reverse	5’- GCCTTGAGCACCAGTTTG − 3’	
*β-actin*	Forward	5’- CACCATTGGCAATGAGCGGTTC − 3’	[29]
	Reverse	5’- AGGTCTTTGCGGATGTCCACGT − 3’	

### ROS detection

ROS generation was assessed with 5,6-chloromethyl-2’,7’-dichlorodihydrofluorescein diacetate (CM-H_2_DCF-DA, Invitrogen). After treating cells with the different treatment groups for 24 h, cells were washed and incubated with 10 µM CM-H_2_DCF-DA (100 µL) for 30 min at 37 °C. Subsequently, excess CM-H_2_DCF-DA was washed, and finally, ROS was detected by a plate reader at 485 nm (excitation) and 530 nm (emission).

### Cell line proliferation assay by BrdU incorporation

For quantification of cell proliferation, BrdU (Roche Molecular Biochemicals) was used. Pre-performing the proliferation assay, cell lines were cultured and incubated with the various pharmacological compounds for 24 h in a 96-well plate at the concentration of 20,000 cells/well. Subsequently, 20 µl of BrdU labeling solution was added (final concentration 10 µM BrdU) and cells were incubated for 24 h at 37 °C. Next, cells were centrifuged at 300 ×g for 10 min, and the upper solution was removed. Cells were then dried by a hair dryer for 15 min. After that, 200 µl/well of FixDenant was added (incubation for 30 min at 20 °C). FixDenant solution was then removed and 100 µl of Anti-BrdU-POX solution was added and cells were incubated for 90 min at 20 °C. Next, the washing solution was used to remove the antibody, and thereafter, washing was removed by flicking it off. Eventually, 100 µl/well of substrate solution was added and the plate was incubated at 20 °C for 20 min. The blank was 100 µl of culture medium.

### Gene knockdown verification at the protein level by colorimetry

To evaluate the protein levels of target genes after 72 h of treatment with the various pharmacological groups, a QuantiSir™ Gene Knockdown Quantification Kit (EPIGENTEK, US) was used. The protocol provided by the manufacturer was followed. Horseradish peroxidase was used as the color developing agent. GAPDH was used as control. The absorbance values were read at 450 nm on a microplate reader [30].

### Cytotoxicity and cell death analysis

Post-treatment with various combinational compounds to assess cell growth, an MTT test was carried out. Primary cells and cell lines (2 × 10^4^ cells/well) were inoculated in a 96-well plate and incubated at 37 °C, 5% CO_2_ for a day. Then, the medium was replaced with 100 µL of medium enriched with various pharmacological mixtures and was incubated for 24–48 h. Next, the medium was replaced with 200 µL of medium containing 10 µL of MTT solution (incubation for 4 h). Finally, 200 µL of DMSO replaced the medium (mixture for 10 min). Eventually, a microplate reader was utilized to assess the OD of each well at 570 nm. The following equation was used to assess the viability percentage of cells [31]:

$$viability=\frac{\left(OD\,treated\,well\left(-blank\right)\right)}{\left(mean\,OD\,control\,well\right(-blank)} \times 100$$

### Evaluation of intrinsic apoptosis pathway by Caspase-9 activity assessment

To assess the activity of Caspase-9, a Caspase-9 Colorimetric Assay (R&D Systems) was used. Following the treatment of cells with various pharmacological groups for 24 h, cells were washed with PBS. Subsequently, cells were lysed with lysis buffer and the cell lysate was used to assess the activity of Caspase-9 according to the directions of the manufacturer. The system measures the level of caspase-9 enzymatic activity in the cell lysate by measuring the color reaction following the addition of a caspase-specific peptide conjugated to a color reporter molecule p-nitroalanine (pNA), which is then cleaved by caspase-9 activity. The OD of the plates was read using a microplate reader at 405 nm.

### Statistical analysis

GraphPad Prism V9 software was used to statistically analyze the data. A one-way ANOVA test was used to evaluate the differences between groups. The linear regression between the parameters was determined by Pearson’s correlation assay. A significance level was considered less than 0.05. Each assay was carried out in triplicate.

## Results

### NPs loaded with Ara-c and siRNAs significantly suppressed HO-1 and HIF-1α expression in AML primary cells and cell lines

**Fig. 2:**
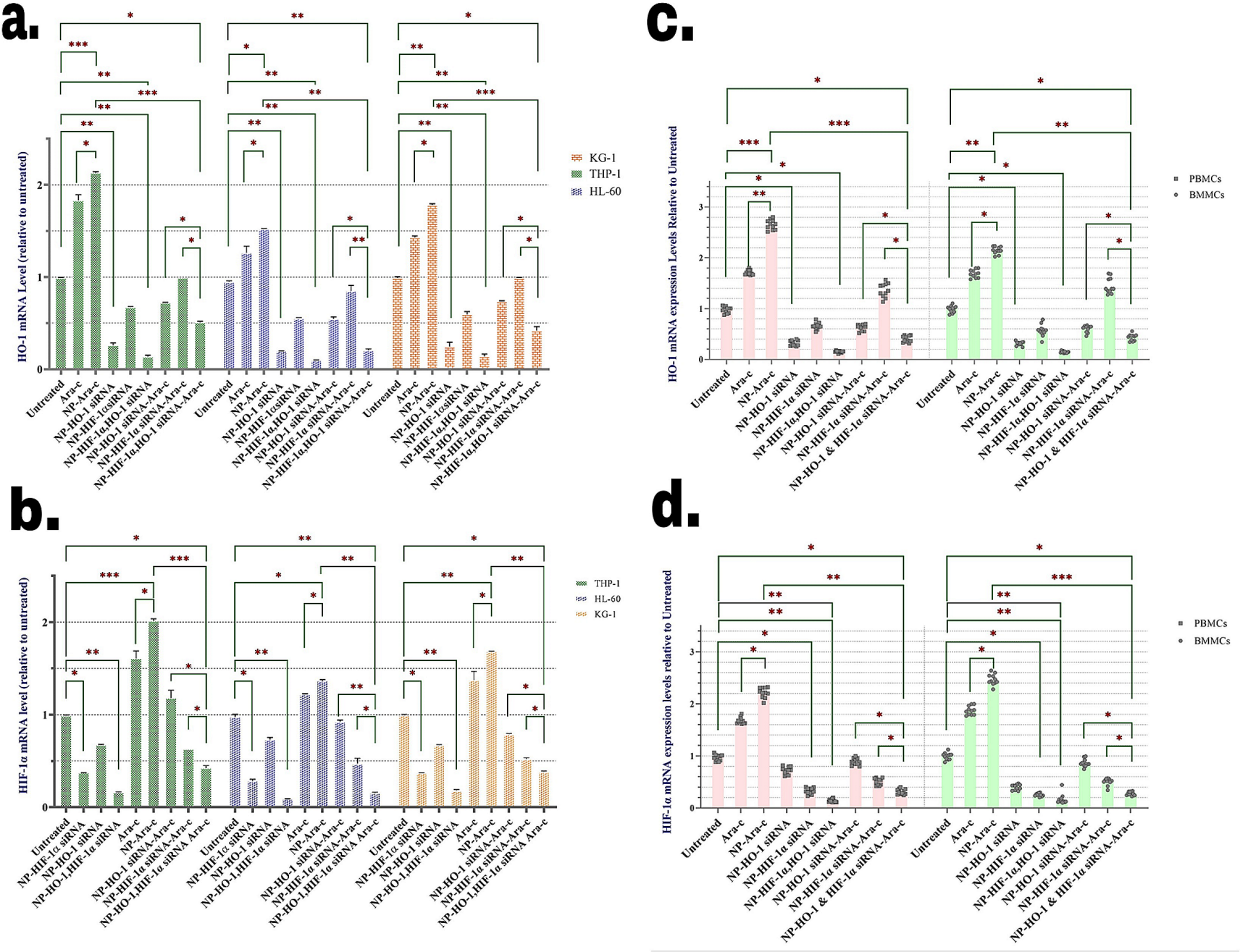
NPs efficiently decreased HO-1 and HIF-1α mRNA levels in treated cells, measured by qRT-PCR. mRNA expression levels of HO-1 after treatment with various pharmacological treatments in (**a**) HL-60, KG-1, and THP-1 cell lines and patients-derived PBMCs and BMMCs (**b**). mRNA expression levels of HIF-1α after treatment with various pharmacological treatments in (**c**) HL-60, KG-1, and THP-1 cell lines and patients-derived PBMCs and BMMCs (**d**). The values are normalized to the β-actin mRNA expression level, and then relatively targeted gene mRNA levels were determined by taking the ratio to the value of the untreated cell as a calibrator based on the ΔΔCT method. The cell line is presented as means ± SD of triplicate experiments. Primary AML patient PBMCs and BMMCs sample data are presented as means of individual patient experiments (*n* = 11). *P*-values *<* 0.05 (*), *P*-values *<* 0.01 (**), and *P*-values *<* 0.001 (***)

qRT-PCR and protein analytic tests were utilized to evaluate the effect of NPs loaded with Ara-c and siRNA molecules on HO-1 and HIF-1α expression in AML PBMCs, BMMCs, and cell lines. Following 24 h treatment of the cell lines and primary cells with NPs loaded with HO-1, HIF-1α, and Ara-c, cellular mRNA was extracted and analyzed. The results demonstrated that following HO-1 silencing by siRNA-loaded NPs, HO-1 mRNA levels were highly reduced in the AML-related cell lines (Fig. 2a) and patient-derived BMMCs and PBMCs (Fig. 2b). Similar results were observed regarding the expression level of HIF-1α in cell lines (Fig. 2c) and primary cells (Fig. 2d) of AML patients following treatment with therapeutics-loaded NPs.

Moreover, the decrease in the expression of each of the factors could have a slight decreasing effect on the expression of the other factor. This result suggests that probably HIF-1α and HO-1 influence each other’s expression. Interestingly, treatment of cells with Ara-c and especially NPs loaded with Ara-c considerably elevated HO-1 and HIF-1α expression. But as expected, treating cells with NPs loaded with siRNAs against HO-1 and HIF-1α could neutralize the inducing effect of Ara-c.

**Fig. 3:**
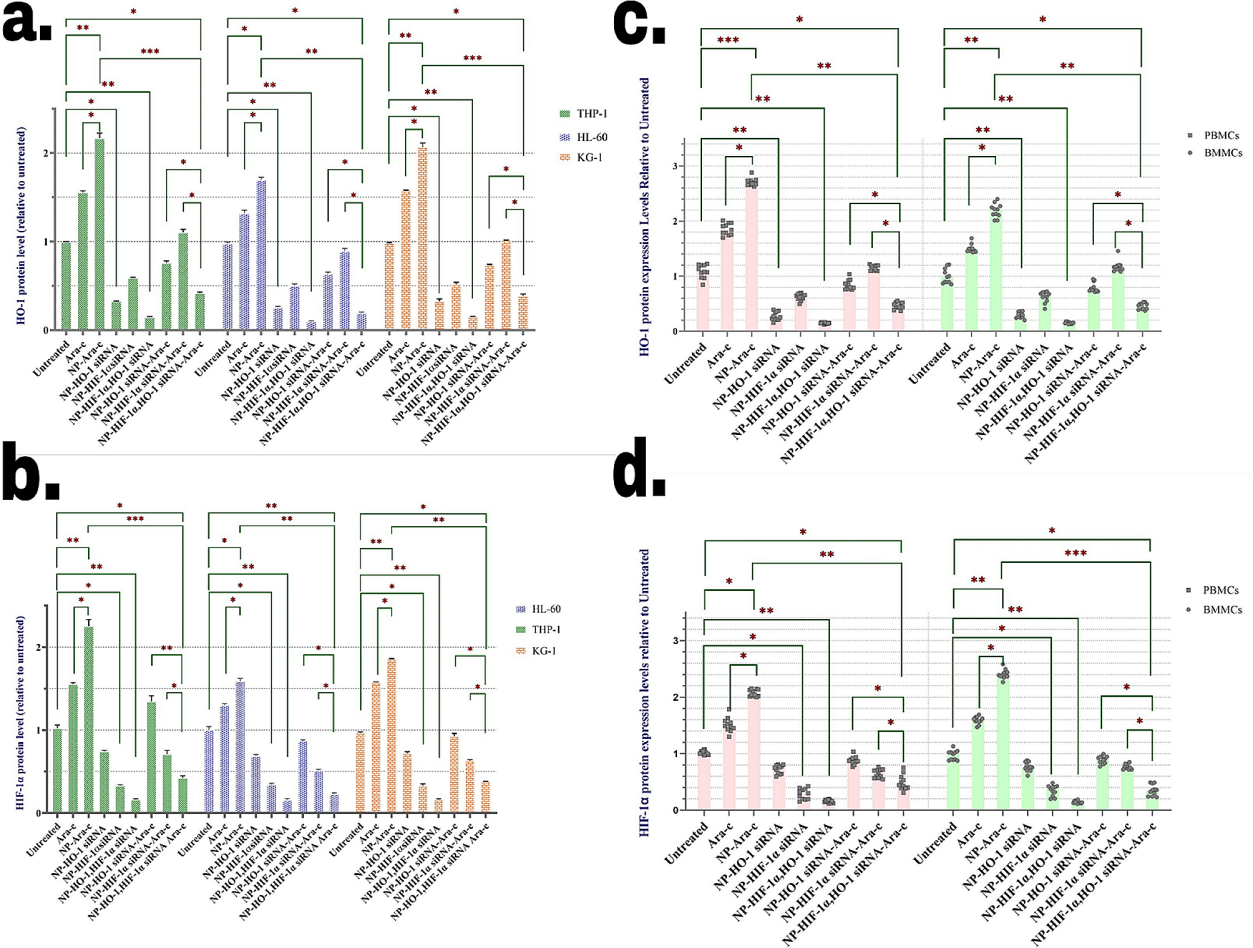
Treatment with NP-bound siRNA inhibited the protein expression of HO-1, and HIF-1α, in AML cells, determined by colorimetry. The protein expression level of HO-1 based on the colorimetric test after treatment with various compounds in (**a**) HL-60, KG-1, and THP-1 cell lines and (**b**) in AML patients derived PBMCs and BMMCs. The protein expression levels of HIF-1α based on the colorimetric test after treatment with various compounds in (**c**) HL-60, KG-1, and THP-1 cell lines and (**d**) in AML patients derived PBMCs and BMMCs. The values are normalized to the GAPDH protein expression level, then relative targeted genes’ protein levels were determined by taking the ratio to the value of the untreated cell as a calibrator. The cell line is presented as means ± SD of triplicate experiments. Primary AML patient PBMCs and BMMCs sample data are presented as means of individual patient experiments (*n* = 11). *P* values < 0.05 (*), *P* values < 0.01 (**), and *P* values < 0.001 (***)

In addition to qRT-PCR, following 72 h of cell incubation with any of the treatments, protein levels were analyzed to evaluate the efficacy of gene silencing (Fig. 3). It was discovered that the level of the HO-1 protein level considerably decreased after exposure of AML cell lines (Fig. 3a) and primary cells (Fig. 3b) to NPs loaded with HO-1 siRNA. Silencing HO-1 also decreased the expression of HIF-1α very slightly. In addition, similar to what was observed in mRNA level expression, treatment of cells with Ara-c and especially NPs loaded with Ara-c significantly increased the expression of HO-1, which was neutralized by the addition of anti-HO-1 siRNA.

Evaluation of HIF-1α expression after treatment with NPs had similar results to HO-1. NPs loaded with anti-HIF-1α siRNA significantly reduced the expression of this factor (this effect was also observed to some extent on HO-1 expression as well) in both cell lines and primary cells of AML patients. Also, Ara-c considerably increased the expression of HIF-1α, which was reversed by adding anti-HIF-1α. (Figs. 3d, e). An interesting point was that the simultaneous addition of both siRNAs caused the greatest silencing effect on both HO-1 and HIF-1α expression in the cells.

### Inhibition of HO-1 and HIF-1α sensitized AML cells to Ara-c in

To determine the IC_50_ value of Ara-c in cell lines and primary cells of AML patients, an MTT test was performed following 24 h incubation with different concentrations of Ara-c. IC_50_ values are presented in Supplementary Figure S1 and Table 1.

**Fig. 4:**
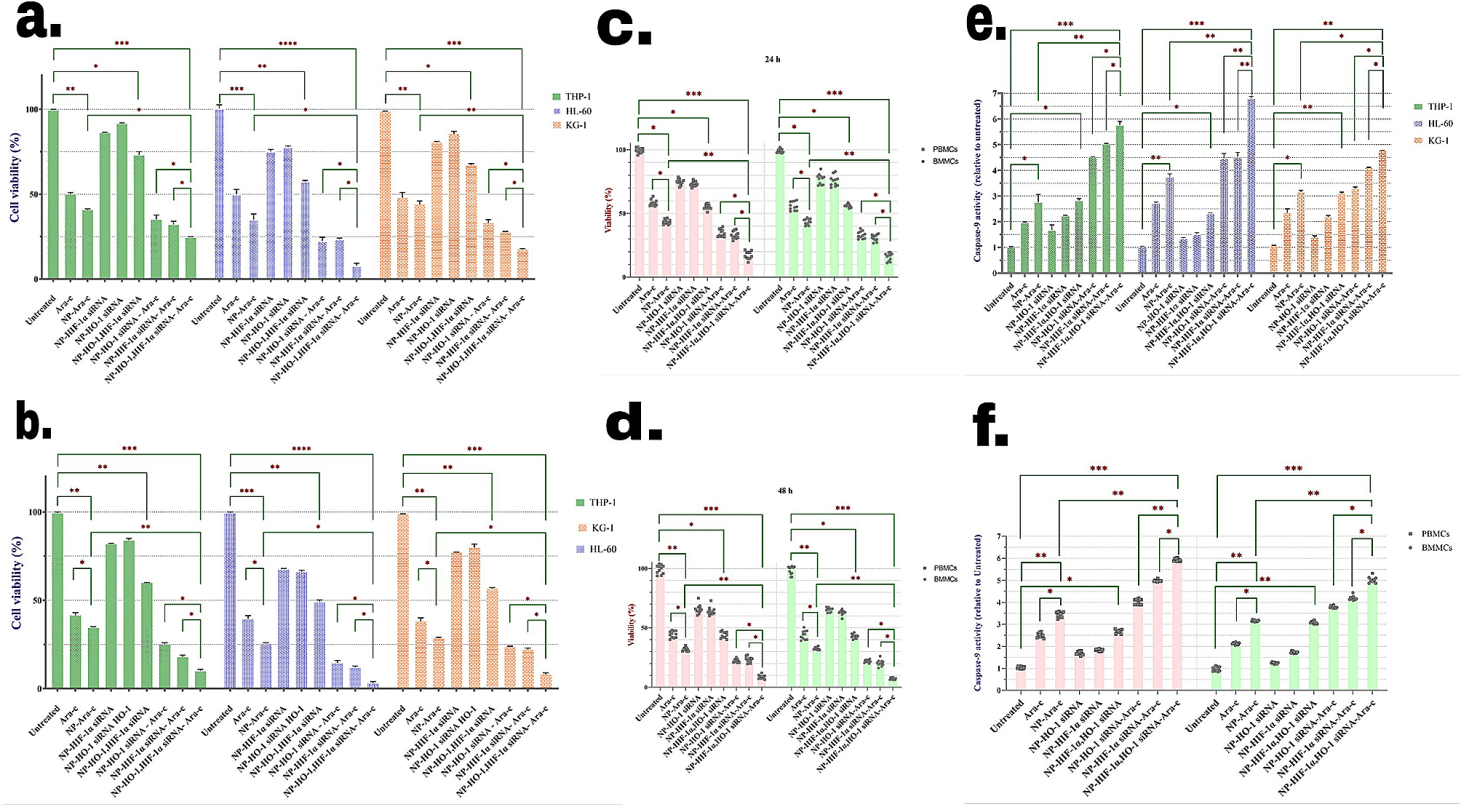
The viability of (**a, b**) HL-60, KG-1, and THP-1 cell lines and patient-derived PBMCs and BMMCs (**c, d**) was decreased following 24 and 48 h treatment with different pharmacological groups. A Caspase-9 assay kit was used to evaluate the activity of Caspase-9 in HL-60, KG-1, and THP-1 cell lines (**e**) and Patient-derived AML cells (**f**) following the treatment with various combination treatments. Cell line data are presented as means ± SD of triplicate experiments. Primary AML patient PBMCs and BMMCs samples data are presented as means of individual patients experiments (*n* = 11). *P*-values *<* 0.05 (*), *P*-values *<* 0.01 (**), and *P*-values *<* 0.001 (***)

Subsequently, based on IC_50_ results, another MTT test was carried out to study the effect of combinational treatments of HO-1/HIF-1α siRNA with Ara-c on the growth of AML cells. After 24 h of cell treatment with the mentioned compounds, we found that inhibition of HO-1 and HIF-1α in combination with Ara-c had an almost similar effect and increased cell sensitivity to Ara-c. Notably, the effect of HIF-1α inhibition was slightly higher when compared to HO-1 silencing. Moreover, the co-inhibition of HO-1 and HIF-1α, induced almost the same apoptosis rates as free Ara-c treatment did. However, the highest rate of cell death was observed in cells that received NPs loaded with siRNAs against HIF-1α, HO-1, and Ara-c treatment. This indicates that inhibition of these two genes could eliminate resistance to Ara-c chemotherapy. Also, it was evident that Ara-c-loaded NPs exerted more apoptosis induction compared to any siRNA-carrying NPs and even higher than free Ara-c. In addition, the growth inhibition was slightly higher in HL-60 cells, compared to KG-1, and THP-1 (Fig. 4a). Also, the effect of treatments increased over the next 24 h incubation, indicating the time-dependency of the treatment (Fig. 4b). The same results were observed in patient-derived AML cells as well (Fig. 4c, d).

### Simultaneous HO-1 and HIF-1α silencing in combination with Ara-c-induced intrinsic apoptosis pathway

To investigate the impact of combination therapy on the intrinsic apoptosis pathway, the activity of Caspase-9 was evaluated in both cell lines and primary cells following 24 h incubation with the treatments (Fig. 4e, f). In cells with silenced HO-1 or HIF-1α genes, the activity of caspase-9 was significantly elevated compared to the controls while, co-inhibition of HO-1 and HIF-1α increased caspase-9 activity almost as free Ara-c, but not as much as NP-Ara-c. Moreover, silencing each gene seemed to form a synergism with Ara-c in inducing Caspase-9 activity. The effect of HIF-1α silencing was slightly higher than HO-1 inhibition. Moreover, it was evident that the co-inhibition of HO-1 and HIF-1α combined with Ara-c treatment was associated with the highest activity of Caspase-9 compared to other cell groups.

### HO-1 and HIF-1α co-inhibition affects the expression level of apoptosis-associated proteins

To study the impact of combination therapy on apoptosis-involved factors, we next evaluated the effect of various treatments on the mRNA levels of apoptosis-related proteins including B-cell lymphoma-2 (Bcl-2) and Bcl-2 Associated X protein (BAX).

**Fig. 5:**
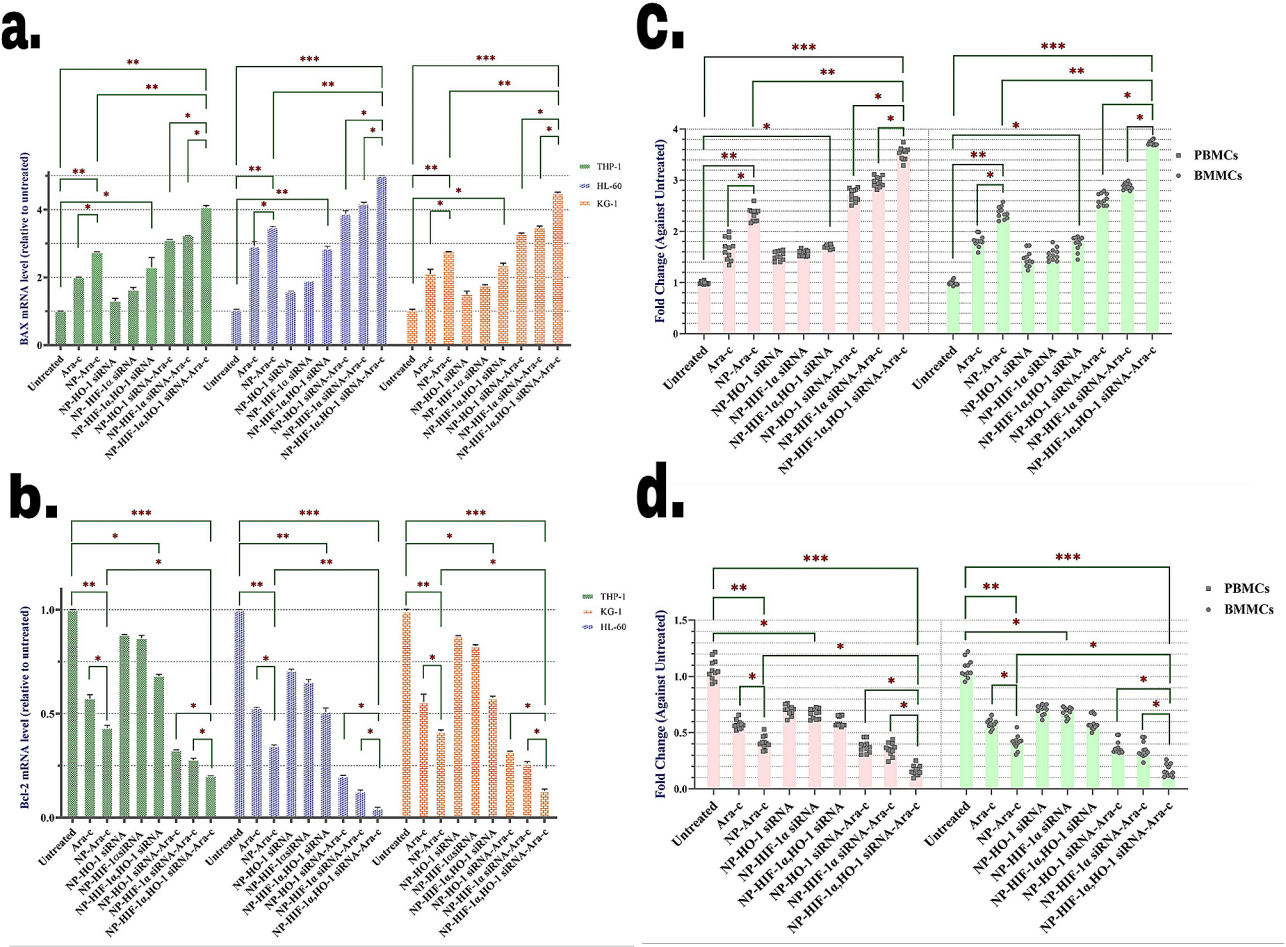
Treating cells with different mixtures of drugs and siRNAs, affected the mRNA expression levels of Bcl-2 and BAX, achieved by qRT-PCR. mRNA expression levels of BAX and Bcl-2 gene analyzed by RT-PCR after 24 h treatment with various combinations in (**a, b**) HL-60, KG-1, and THP-1 cell lines and patient-derived PBMCs and BMMCs (**c, d**). The values are normalized to the β-actin mRNA expression level, and then the relatively targeted genes’ mRNA levels are determined by taking the ratio to the value of the untreated cell as a calibrator based on the ΔΔCTT method. Cell lines data are presented as means ± SD of triplicate experiments. Primary AML patient PBMCs and BMMCs samples data are presented as means of individual patients experiments (*n* = 11). *P*-values *<* 0.05 (*), *P*-values *<* 0.01 (**), and *P*-values *<* 0.001 (***)

Treating cell lines with HIF-1α siRNA-NP significantly decreased Bcl-2 mRNA expression and elevated the expression of BAX mRNA as for the treatment with HO-1 siRNA-NP but with slighter amounts (Fig. 5a, b). The same results were observed with primary cells as well (Fig. 5c, d). Co-inhibition of HIF-1α and HO-1 exerted a more significant effect which was even comparable with free Ara-c treatment. On the other hand, the effect of NP-Ara-c was much more considerable. The results demonstrated a notable increase in changes of Bcl-2 and BAX mRNAs following the treatment with NPs loaded with HO-1 and HIF-1α siRNAs and Ara-c. Thus, silencing these two genes directly induced apoptosis in AML cells.

### Codelivery of anti-HIF-1a/HO-1 siRNAs and Ara-c induced oxidative stress in leukemic cells

**Fig. 6:**
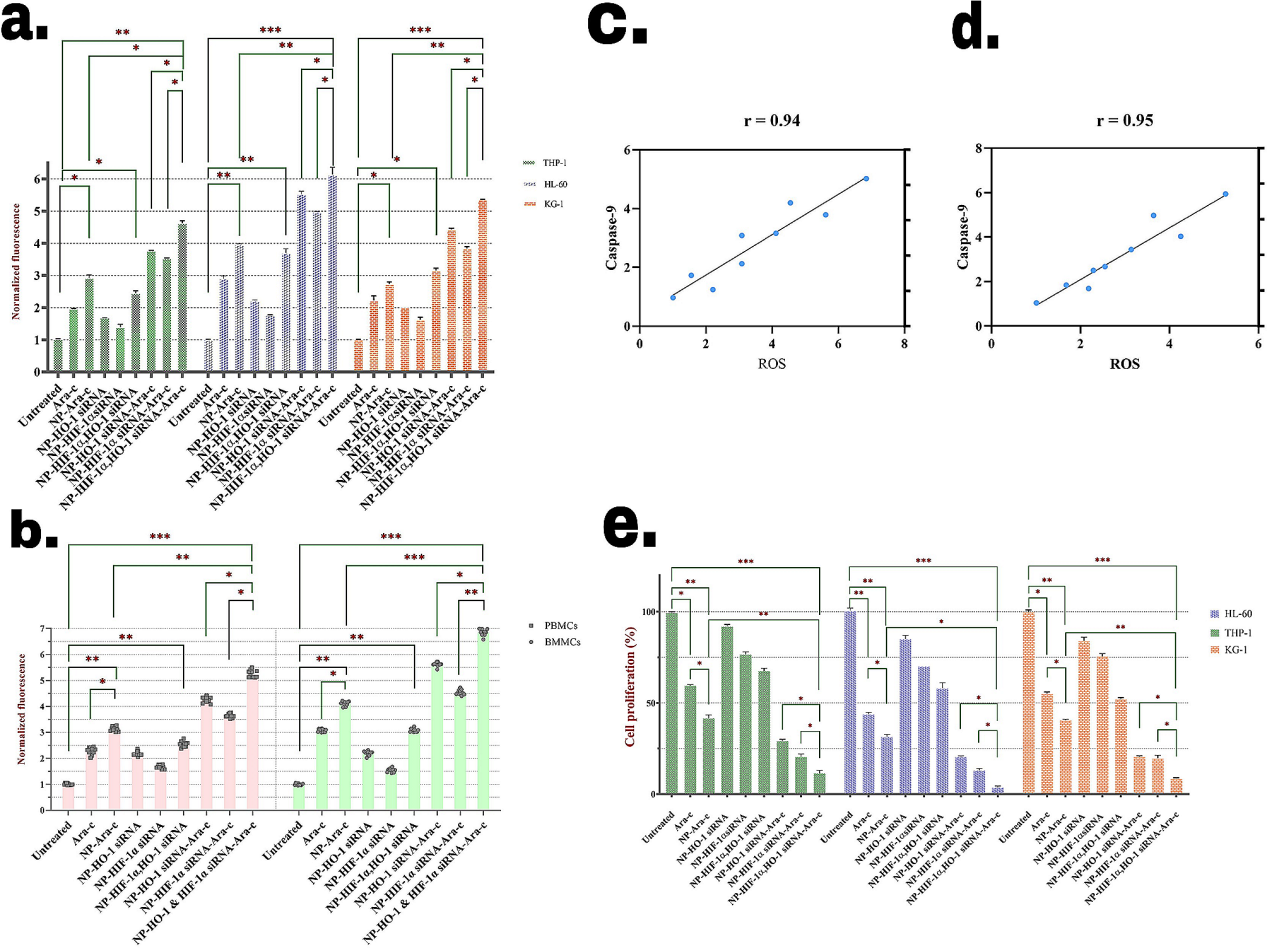
Treating cells with different pharmacological groups affects ROS formation as well as the proliferation potential to. The effect of 24-hour treatment with various treatment groups on ROS production was measured by DCF-DA in HL-60, KG-1, and THP-1 cells (**a**) and patient-derived PBMCs and BMMCs (**b**). Correlations between caspase-9 activity and the ROS levels in BMMCs (**c**) and PBMCs (**d**) upon exposure to various treatments. Blue dots are the means of 11 replicates. Pearson’s correlation coefficients are indicated on each plot. The solid line illustrates the regression line. (**e**) The result of single and combinational treatments on the proliferation of AML cell lines following 24 h incubation. Data were means ± SD (*n* = 3); *P < 0.05, **P < 0.01, ***P < 0.001, compared to untreated cells

We also evaluated the effect of treatments on the production of ROS in cell lines (Fig. 6a) and primary cells (Fig. 6b), as Ara-c is a strong inducer of ROS generation in AML cells leading to cell apoptosis.

The results showed that NP-Ara-c treatment induced higher levels of intercellular ROS compared to free Ara-c treatment. In addition, inhibition of HO-1 and HIF-1α increased the generation of ROS as well. The effect of HO-1 inhibition was slightly higher compared to HIF-1α silencing. Silencing of each gene synergistically induced ROS generation with Ara-c treatment. The highest levels of ROS generation were observed in cells treated with NPs loaded with siRNAs against HIF-1α and HO-1 and Ara-c.

Regarding the association of ROS production and apoptosis, we then evaluated the correlation between ROS generation and caspase-9 activity in AML primary cells (Fig. 6c and d).

As demonstrated by the graphs, a very strong correlation (*r* = 93, *r* = 94) exists between ROS production and caspase-9 activity in AML PBMCs as well as BMMCs, indicating that ROS generation is a major contributor to programmed cell death of AML cells.

### The combinational therapy inhibited the proliferation of AML cells

The proliferation of AML cell lines in response to the various combinational treatments was also assessed (Fig. 6e). Treating cells with each siRNA exerted a slight effect on the proliferation of cells, while treatment with both siRNAs inhibited proliferation considerably, which was comparable with free Ara-c treatment. Another finding was that the effect of HIF-1α inhibition on proliferation was slightly higher compared to HO-1 inhibition. Moreover, compared to treatment with free Ara-c, cells treated with NP-Ara-c demonstrated a more significant inability to proliferate. The inhibition of each gene seemed to form a synergism with Ara-c to inhibit proliferation. The highest level of proliferation inhibition was noted in the group receiving both siRNAs and Ara-c-loaded NPs.

## Discussion

AML as one of the main hematological malignancies is associated with frequent relapses and poor prognosis, especially in the elderly despite the use of intensive chemotherapies [5].

In addition to relapse, chemoresistance is another clinical issue emerging post-AML treatment [32].

Moreover, Ara-c/DNR (the main chemotherapy regimen for AML) doses are highly toxic and have plenty of side effects [33]. Thus, the need for novel therapeutic approaches to reverse this drug resistance and reduce the required dose of chemotherapy to efficiently kill tumor cells is always felt.

Nowadays, combination therapy and simultaneous manipulation of two genes is a very common method in such studies. A large part of the studies investigate the effect of silencing several genes together and alone, to compare the effect of simultaneous inhibition of the genes with inhibition alone [34]. Combination gene therapy cooperates with chemotherapy to ensure the maximum death of tumor cells and decrease the possibility of relapse. Combination gene therapy overcomes the limitations of chemotherapy, including multidrug resistance, high drug dose, side effects, etc. To be able to successfully use combination gene therapy in clinics, certain issues need to be solved. First, the long-term effects of this treatment must be studied thoroughly to ensure its safety. Second, safe vectors are required to efficiently deliver the cargo to the target cells. This makes nanoparticles a new promising tool for this aim [35]. Therefore, combined gene therapy with chemotherapy strategy was investigated on AML cells in this study.

HO-1 is an inducible enzyme, that catabolizes heme into biliverdin, iron, and carbon monoxide [36].

According to several studies, HO-1 is reported to be upregulated in AML cell lines and primary cells of AML patients. The overexpression of HO-1 in AML patients is associated with poor prognosis [6, 37, 38]. HO-1 promotes resistance to chemotherapy using a variety of pathways. Neutralizing ROS [6], induction of the JNK/c-jun pathway to inhibit apoptosis [38], and many other mechanisms are examples of HO-1 anti-apoptotic functions. Moreover, HO-1 can exert cytoprotective activities by its products [39], including the induction of ferritin heavy chain transcription and Fe efflux pump function, induced by Fe^2+^ atom released from catabolized heme by HO-1, resulting in decreased ROS formation [40–43]. In addition to Fe, biliverdin, which is then converted to bilirubin ( a strong antioxidant molecule) can protect tumor cells from ROS, and also the anti-inflammatory effects of bilirubin (due to its effect on inhibiting the expression of P and E selectins) [44–46] are several mechanisms to name a few. In addition to leukemic cells, HO-1 is reported to be overexpressed in CD34^+^CD38^-^ as well as CD34^+^CD38^-^ cells (both stem cells and progenitor cells) of AML patients compared to normal CD34^+^ cells, which brings to mind the idea of possible benefit of HO-1 silencing in eliminating the Minimal Residual Disease (MRD) [3].

HIF-1α is a well-known regulator of angiogenesis and proliferation. HIF-1α is degraded in normoxic conditions by the ubiquitin proteasomal system due to proline residue hydroxylation, while preserved from degradation in hypoxic conditions [47, 48]. However, this is not completely certain. Since it has been shown that in addition to hypoxia, other molecular mechanisms can regulate the levels of this protein in even normal cells [49]. In normal cells, HIF-1α is proven to be activated by the signaling of Phosphoinositide 3-kinase activity/ protein kinase B (PI3K/AKT) [50, 51], and CDK2 activity, through blockade of lysosomal HIF-1α degradation in normoxia [52]. Additionally, CDK5 activity has proven to elevate HIF-1α protein levels in cells by stabilizing phosphorylation of HIF-1α at Ser687 mediated mechanism [53]. Also, it has been reported that HIF-1α can be stabilized by a ROS-mediated mechanism in normoxic conditions [54]. Besides oxygen-independent HIF-1α stabilization in normal cells, under oncogenic mutation or inactivation of anti-oncogenes, HIF-1α can be activated under normoxic conditions as well [55–57]. Mutations of oncogenic genes including *P53, RB, Ras, ARF*, and *Myc* are proven to stabilize HIF-1α in cancer cells [58].

In AML in particular, several other mechanisms have also been proposed to explain the reason behind the accumulation of HIF-1α in cells in normoxia. In 10–15% of AML cases, the mutations of Isocitrate Dehydrogenase (IDH) 1 and 2 result in PHD inactivation, therefore the suppression of HIF-1α hydroxylation [59]. Moreover, FMS-Like Tyrosine kinase 3 (FLT3) mutations which are found at a frequency of 25–355 in AML, activate PI3K/AKT in the cells [60, 61], resulting in the activation of Mammalian Target Of Rapamycin (mTOR) and subsequently the accumulation of HIF-1α in the cells [62, 63]. Additionally, Nucleophosmin (NPM1) mutations (observed in 30–40% of AML cases) can induce P14ARF degradation, which in turn stimulates HIF-1α activity [59]. Finally, the accumulation of HIF-1α in AML cells could be due to the P53 loss of function (observed in 10% of AML) resulting in decreased expression of Mouse double minute 2 homolog (MDM2 which is an alternative E3 ubiquitinylation ligase for HIF-1α [59].

Therefore, HIF-1α is overexpressed in many cancers and AML [17, 64–66]. HIF-1α induces the expression of many genes related to chemoresistance, including GLUT-1, HO-1, VEGF, etc [1, 18]. , making HIF-α an ideal target for cancer therapy [67].

Based on several studies, HO-1 and HIF-1α are overexpressed in AML cells. This overexpression is associated with resistance to DNR, and Ara-c [1, 2, 17, 20]. Based on another study, HIF-1α is overexpressed in AML cells that possess mutant TP53 gene (an indicator for poor prognosis in AML, and poor response to chemotherapy) compared to AML cells with wild TP53 gene (an indicator of ideal prognosis) [68].

For the reasons stated above, as well as the proven role of both molecules in various cancers, we selected these two genes to assess the effect of their simultaneous inhibition in response to Ara-c treatment, a main chemotherapy regimen used for AML treatment. To silence the expression of these genes, we used siRNA molecules. Due to the instability of siRNA in the extracellular environment, we used NPs to transport siRNA into cells. The usage of NPs to deliver drugs is very efficient in chemotherapy [69].

In this study, TAT-CCMD-PEG-NPs were utilized to transport siRNA and Ara-c to the cells. The usage of CCMD improves the loading and delivery of NPs, while binding NPs via PEG to TAT, increases the cellular uptake by cells. We designed these NPs and performed all the required tests to characterize TAT-CCMD-PEG-NPs (including transfection and cargo delivery efficiency and other required tests as well) in our recent research [22].

Based on the MTT results, it can be seen that following the silencing of HO-1, the rate of growth in response to Ara-c treatment was greatly decreased. The reason behind this sensitivity following the inhibition of HO-1 may be due to the fact that Ara-c induces ROS production, which is lethal to the cell. But this ROS is neutralized by the overexpressed HO-1 (induced by high ROS levels), which is why cell treatment with Ara-c induces HO-1 expression, and HL-60R cells that are resistant to Ara-c have higher levels of HO-1 expression [1]. Thus, we evaluated the level of ROS generated following the inhibition of each gene as well as the treatment with Ara-c. We found that HO-1 inhibition induced the repletion of ROS in AML cells following Ara-c treatment.

HIF-1α inhibition also induced ROS generation, (probably because HIF-1α regulates HO-1 expression as well as other stress responder genes, and its silencing can affect the expression of these genes) but to a lesser amount. The highest level of ROS was observed following the inhibition of both genes and treatment with Ara-c.

Following the inhibition of the *HIF-1α* alone, the rate of growth inhibition was higher than in the case in which the HO-1 gene was silenced. This is probably due to the fact that HIF-1α controls more genes that are involved in Ara-c resistance. However, the highest rate of apoptosis was seen when the two genes were inhibited simultaneously by NPs and cells were treated via Ara-c. We further studied the role of these genes in preventing cell apoptosis by studying the effect of HO-1 and HIF-1α silencing on apoptosis-related genes including Caspase-9, Bcl-2, and BAX. Inhibition of these two genes decreased Bcl-2 and increased BAX expression. This indicates the role of HO-1 and HIF-1α in preventing apoptosis. Following the inhibition of either of the genes, the activity of Caspase-9 increased. Therefore, the co-inhibition of HIF-1α and HO-1 synergizes with Ara-c treatment and activates the intrinsic apoptotic pathway. The effect of HIF-1α inhibition was slightly higher than HO-1 inhibition, this may be also due to the regulation of several anti-apoptotic genes by HIF-1α. Our study indicates that HO-1 and HIF-1α contribute to Ara-c resistance in AML, and silencing them could overcome this resistance possibly by increasing ROS levels in AML cells, thus resulting in apoptosis.

Based on Pearson’s correlation results, a positive connection was noted between caspase-9 concentration and ROS levels in the cells, following the treatment with various combinational treatment groups, indicating that the elevated levels of ROS in cells induced by HO-1 and HIF-1α silencing as well as treatment with Ara-c, contribute in the induction of caspase-9-dependant apoptosis. Therefore, a major mechanism synergistically being used to induce apoptosis by HO-1, HIF-1α silencing, and Ara-c treatment is the induction of ROS generation which in turn results in caspase-dependent cell death. However, several other mechanisms may be of importance as well.

We next evaluated the effect of treatment on the proliferation of cell lines and found that HIF-1α and HO-1 co-silencing could exert almost the same effect as free Ara-c did. Moreover, the effect of HIF-1α inhibition was slightly higher compared to the HO-1 inhibition result. However, cells with silenced HO-1 and HIF-1α combined with Ara-c treatment demonstrated the lowest level of proliferation.

Silencing both genes significantly improved apoptosis induction. Silencing HO-1 induced higher levels of ROS compared to HIF-1α silencing, while the data regarding the proliferation of AML cells was vice-versa.

Our results are in line with a study indicating that HO-1 inhibition sensitizes cells to Ara-c treatment. It is suggested that HO-1 silencing could sensitize AML cells to Ara-c by increasing intercellular ROS levels [1]. Based on the results of another study, transfection of AML-M2 cells with HO-1 siRNA decreased the viability of AML cells and improved the survival of AML mice models in response to DNR treatment [2]. Zhe et al. also reported that following the inhibition of HIF-1α by 2-methoxyestradiol, the survival, and proliferation of AML cells decreased more significantly compared to cells treated with Ara-c. They also showed that HIF-1α inhibition by this agent eliminated AML stem cells more efficiently than Ara-c. They found that this HIF-1α inhibitor induces ROS generation, which then triggers the mitochondrial apoptotic pathway [17].

Wang et al. also reported that treatment of *TP53* mutated AML cells with echinomycin (a HIF-1α inhibitor) eliminates AML stem cells more efficiently than DNR + Ara-c treatment. Inhibition of HIF-1α also improved the survival of TP53-mutated AML murine models as well. Thus, HIF-1α inhibition could be beneficial even in eliminating MRD, due to its potential for selective elimination of leukemic stem cells [68].

Future studies can evaluate the effect of this combinational therapy with a higher number of patients, and compare the results in T-ALL and B-ALL groups, which was not possible in the current study due to the scarcity of samples. Moreover, the effect of this treatment on other cellular processes for example cell cycle, metabolism, etc. should be further studied as well. In the second step, the treatment could be used in AML xenograft models to help provide a more practical result and also evaluate. Finally, clinical trials could prove the applicability of the treatment in practice as well.

## Conclusions

As far as we know, this was the first time that HO-1 and HIF-1α were simultaneously silenced using NPs loaded with siRNA to evaluate its effect on Ara-c chemoresistance. To sum up, while HO-1 mostly induces resistance to ROS, HIF-1α is involved in the induction of cell proliferation. Therefore, both genes contribute to chemoresistance and cosilencing of HO-1 and HIF-1α eliminates chemoresistance, proliferation potential, induces ROS generation, caspase-9 activity, and thus apoptosis. This study presents new hope for individuals afflicted with AML.

### Electronic supplementary material

Below is the link to the electronic supplementary material.

Supplementary material 1

## Data Availability

No datasets were generated or analysed during the current study.

## Abbreviations

AML: Acute Myeloid Leukemia
Ara-c: Cytarabine
BAX: Bcl-2 Associated X protein
Bcl-2: B-cell lymphoma-2
BM: Bone Marrow
BMMC: Mone Marrow Mononuclear Cells
BrdU: Bromodeoxyuridine
CCMD: Chitosan-Carboxymethyl Dextran
CMDFA: 5,6-chloromethyl-2’,7’-dichlorodihydrofluorescein diacetate
CXCR4: CXC chemokine type 4 Receptor
DNR: Daunorubicin
ELISA: Enzyme-Linked ImmunoAssay
GLUT − 1: Glucose Transporter-1
HIF-1α: Hypoxia-Inducible Factor-1 alpha
HO-1: Heme Oxygenase-1
HSCT: Hematopoietic Stem Cell Transplantation
Hsp32: Heat shock protein 32
MRD: Minimal Residual Disease
MTT: 3-(4,5-dimethylthiazol-2-yl)-2,5-diphenyl-2 H-tetrazolium bromide
NP: NanoParticles
PB: Peripheral Blood
PBMC: Peripheral Blood Mononuclear Cells
PEG: Poly Ethylene Glycol
ROS: Reactive Oxygen Species
SiRNA: Small interfering RNA
TAT: Trans-Activator of Transcription
VEGF: Vascular Endothelial Growth Factor
