# Supplementary material for: The sensitivity of acute myeloid leukemia cells to cytarabine is increased by suppressing the expression of Heme oxygenase-1 and hypoxia-inducible factor 1-alpha
Source: Cancer Cell Int. 2024 Jun 25;24:217. doi: 10.1186/s12935-024-03393-3 (PMC11197338; doi:10.1186/s12935-024-03393-3)
Supplement: Supplementary file 1 — Supplementary material 1 [file 12935_2024_3393_MOESM1_ESM.docx]

Supplementary Table S1. Patients' characteristics

| Sample number | AML  #1 | AML  #2 | AML  #3 | AML  #4 | AML  #5 | AML  #6 | AML  #7 | AML  #8 | AML  #9 | AML  #10 | AML  #11 |
| --- | --- | --- | --- | --- | --- | --- | --- | --- | --- | --- | --- |
| Sample type | BM | BM | BM | BM | BM | PB | BM | PB | BM | PB | PB |
| FAB subtype | M2 | M2 | M2 | M4 | M2 | M4 | M4 | M2 | M5 | M7 | M1 |
| age | 66 | 57 | 70 | 50 | 72 | 69 | 58 | 70 | 56 | 66 | 45 |
| sex | F | M | F | F | F | F | F | F | M | F | F |
| WBC | 126  H | 5.55  H | 7.98  H | 143  H | 3.30  H | 5.79  H | 44.59  H | 9.07  H | 50.45  H | 1.28  H | 7.78  H |
| RBC | 0.76  L | 0.53  L | 0.44  L | 2.08  L | 1.85  L | 2.29  L | 0.35  L | 2.64  L | 0.87  L | 2.33  L | 2.62  L |
| plt | 78  L | 6  L | 16  L | 40  L | 25  L | 46  L | 29  L | 59  L | 25  L | 75  L | 39  L |
| Hb (g/dl) | 2.5  L | 1.6  L | 1.6  L | 6.4  L | 5.6  L | 7.8  L | 1.1 L  L | 7.4  L | 2.4  L | 6.1  L | 7.9  L |
| HCT | 2.5 %  L | 5.5%  L | 5.3%  L | 19.8%  L | 15.9%  L | 23.7%  L | 3.8%  L | 24.9%  L | 8.4%  L | 17.7%  L | 24.5%  L |
| MCV (fL) | 104.0  H | 102.9  H | 122.2 | 95.2 | 86 | 103.7  H | 108.5  H | 94.9 | 97.2 | 76.1  L | 93.7  H |
| MCH (pg) | 33.0  H | 30.5 | 35.5  H | 30.6  L | 30. | 34.0 H | 31.4 | 26.2 | 28.1 | 26.4  L | 30.4  L |
| MCHC (g/dL) | 31.7  H | 29.7  L | 29.0 | 32.1  L | 35.4 | 32.8 | 29.0  L | 29.6  L | 29.9  L | 34.6 | 32.4  L |
| RDW | 18.0 %  H | 21.5%  H | 16.4%  H | 20.0%  H | 17.1%  H | 20.7%  H | 18.7%  H | 19.5%  H | 18.3%  H | 15.8%  H | 16.7%  H |
| NEUT | 48.3% | 43.4%  L | 24%  H | 61.3%  L | 48.4% | 56.9% | 54.8% | 36.7%  L | 5.2%  L | 28%  L | 3.4%  L |
| LYMPH | 29.0%  H | 18.6%  L | 69.4%  H | 6.1%  L | 38.8% | 17.5%  L | 18.2%  L | 34.6% | 9.3%  L | 41.4% | 58.0%  H |
| MONO | 17.0%  H | 29.9%  H | 5.2%  L | 24.7%  H | 10.8%  H | 4.1%  H | 22.5%  H | 16.7%  H | 3.4% | 11.1%  H | 19.6%  L |
| CD13(%) | 76 | 31 | 35 | 34 | 9 | 62 | 64 | 7 | 34 | 5 | 86 |
| CD33(%) | 73 | 88 | 46 | 98 | 24 | 51 | 72 | 74 | 96 | 5 | 91 |
| CD117(%) | 69 | 52 | 35 | 49 | 50 | 30 | 36 | 66 | - | - | 85 |
| CD14(%) | 14 | 3 | 8 | 21 | - | 38 | 38 | - | 41 | - | - |
| CD64(%) | 31 | 33 | - | 75 | - | 40 | 54 | 15 | 96 | - | - |
| CD11b(%) | 0 | 8 | - | 63 | - | 48 | 29 | - | 86 | - | - |
| CD41 | - | - | - | - | - | - | - | - | - | 72 | - |

Abbreviations: Acute Myeloblastic Leukemia (AML), Peripheral Blood (PB), Bone Marrow (BM), White Blood Cell (WBC), Red Blood Cell (RBC), PLT (Platelet), Hemoglobin (Hb), Red blood cell Distribution Width (RDW)


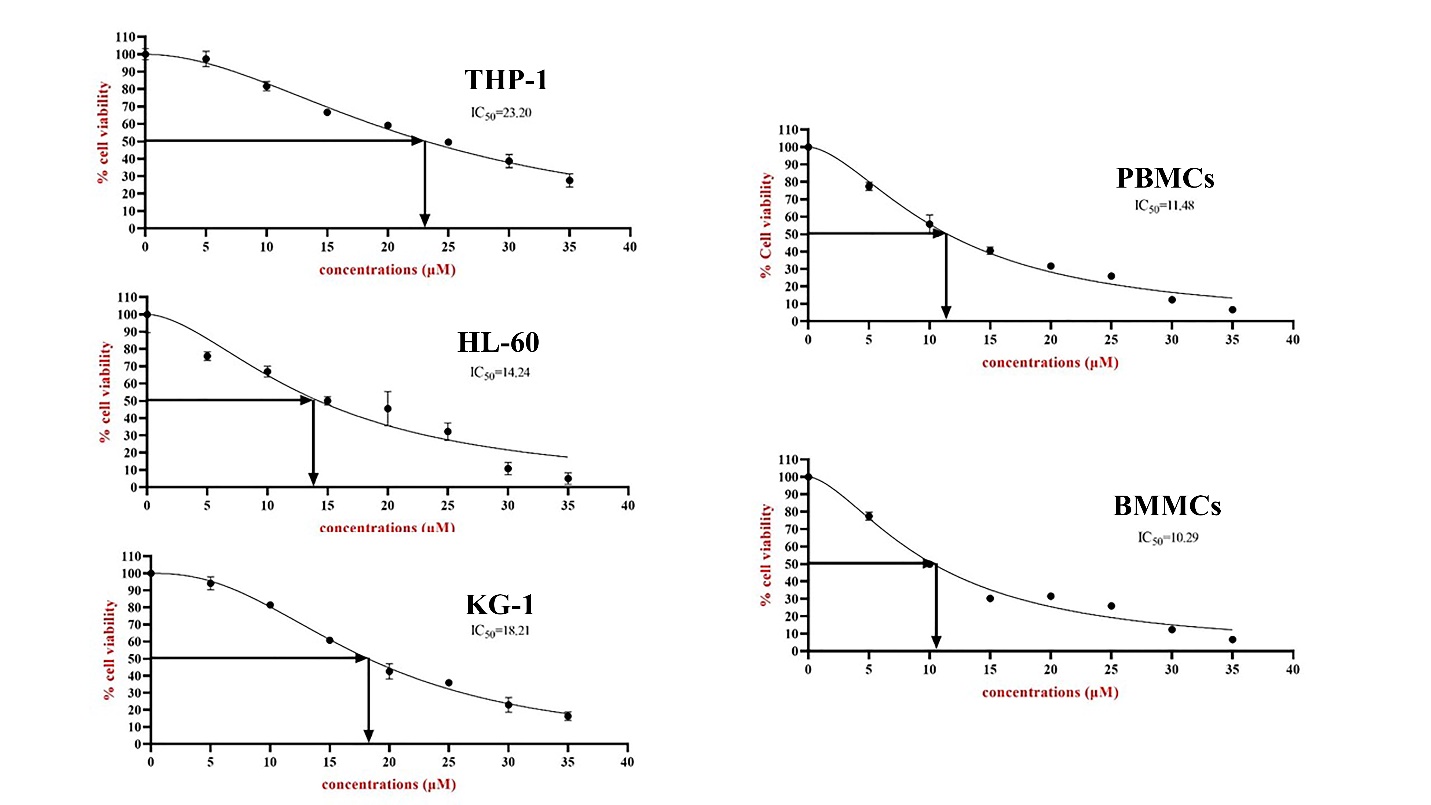


**Figure S1**: The IC_50_ values of Ara-c for AML cell lines (a, b, c) and primary cells (d, e) (n=11) following 24 h treatment.
